# Proteomic and physiological responses in mangrove *Kandelia candel* roots under short-term high-salinity stress

**DOI:** 10.3906/biy-1906-22

**Published:** 2019-10-14

**Authors:** Jianhong XING, Dezhuo PAN, Lingxia WANG, Fanglin TAN, Wei CHEN

**Affiliations:** 1 College of Life Sciences, Fujian Agriculture and Forestry University, Fuzhou, Fujian China; 2 College of Resources and Chemical Engineering, Sanming University, Sanming, Fujian China; 3 College of Life Sciences, Ningxia University, Yinchuan, Ningxia China; 4 Fujian Academy of Forestry Sciences, Fuzhou, Fujian China

**Keywords:** Proteome, energy metabolism, cell wall, antioxidation, triterpenoids

## Abstract

*****Kandelia candel* is one of the mangrove species that are most resistant to environmental stress. As a typical nonsalt-secreting mangrove plant, *K. candel* is an ideal biological material to analyze the molecular mechanism of salt tolerance in woody plants. In this study, changes in protein abundance and expression profile in *K. candel* roots under high-salinity stress of 600 mmol L^-1^ NaCl were analyzed using isobaric tags for relative and absolute quantification (iTRAQ) assay. Moreover, the physiological parameters associated with metabolic pathways in which the differentially abundant proteins (DAPs) are involved were determined. A total of 5577 proteins were identified by iTRAQ analysis of the *K. candel* root proteins, of which 227 were DAPs with a fold change ratio >1.2 or a fold change ratio <0.83 and a P-value <0.05. A total of 227 DAPs consisting of 110 up-regulated and 117 down-regulated proteins were identified. Our Gene Ontology (GO) and Kyoto Encyclopedia of Genes and Genomes (KEGG) pathway analyses revealed that the DAPs were primarily involved in biological processes including carbohydrate and energy metabolisms, stress response and defense, cell wall structure, and secondary metabolism. The results of the physiological parameters showed that their profile changes were consistent with those of the proteome analysis. The results of the proteome and physiological parameters showed that *K. candel* roots could resist high-salinity stress by maintaining a normal Embden-Meyerhof-Parnas and tricarboxylic acid (EMP-TCA) pathway, increasing the activities of various antioxidant enzymes and antioxidant contents, stabilizing the cell wall structure, and accumulating secondary metabolites such as triterpenoids.

## 1. Introduction

Salt stress is one of the primary abiotic stresses that affect plant growth and global crop yields (Miyazaki et al., 2007; Song et al., 2011). A high-salinity environment can cause ionic toxicity, osmotic stress, and oxidative damage to plant cells, affect photosynthetic biological processes, respiration, and energy metabolism, and eventually leads to plant growth arrest or even death. Simultaneously, soil salinization causes a decline in land productivity and seriously threatens the sustainable development of agriculture and forestry (Bandehagh et al., 2011; Kosová et al., 2011). Therefore, it is very important to study the salt tolerance mechanisms of plants, excavate salt tolerance genes, and cultivate new salt-tolerant varieties.

Proteomics technology provides an effective method for studying plant adaptation to abiotic stresses; thus, it has been widely used to understand the molecular mechanisms of plant salt stress (Tuteja, 2007; Soni et al., 2015). Isobaric tags for relative and absolute quantification (iTRAQ) technology developed in recent years can efficiently and accurately detect the changes of protein content, providing an effective technical platform for detailed analysis of molecular mechanisms of plant salt stress responses (Gong et al., 2014; Cheng et al., 2016). Proteomic changes in model plants, major crops and some halophytes under salt stress have previously been analyzed by iTRAQ technique in *Arabidopsis thaliana* and *Thellungiella halophila* (Pang et al., 2010), *Gossypium hirsutum *L. (Li et al., 2015; Gong et al., 2017), *Oryza sativa* L. (Xu et al., 2015a), *Glycine max *cv Dongnong 50 (Ji et al., 2016), maize inbred lines Jing724 and D9H (Luo et al. 2017), *Triticum aestivum *(Jiang et al., 2017), *Musa paradisiaca* (Ji et al., 2019), and *Halogeton glomeratus* (Wang et al., 2016a). These studies demonstrated that photosynthesis, carbohydrate and energy metabolism, signal transduction, membrane transporters, stress responses, and defense all played important roles in response to salt stress in roots and leaves. 

Proteome studies on salt tolerance mechanisms of mangrove plants have also been reported. Zhu et al. (2012) analyzed the proteomic changes of *Bruguiera gymnorhiza* roots under salt stress using 2-DE technique and found that fructose-1,6-diphosphate aldolase (FBP) protein is up-regulated in the primary roots, while an osmotic regulatory protein in the lateral roots was up-regulated at early stages of stress. Therefore, the trend of protein expression was inconsistent with that of the corresponding gene expression. A 2-DE study of proteins in *Avicennia marina *leaves under salt stress showed that nitric oxide (NO) signaling pathway improves salt tolerance by enhancing photosynthesis, energy and primary metabolisms (Shen et al., 2018b). In our previous studies, we investigated the changes in chloroplast proteins in *K. candel* seedling leaves in response to salt stress using iTRAQ technique. It has been proven that photosynthesis, respiration and energy metabolism, signal transduction, osmotic regulation, Na+ compartmentalization, and antioxidant metabolism play important roles in salt tolerance of *K. candel *species**(Wang et al., 2014, 2015, 2016b). 

The mangrove *K. candel* is a type of woody halophyte that grows in the intertidal zones of tropical and subtropical oceans. During a long evolutionary process, *K. candel* formed a unique salt tolerance mechanism which enables it to withstand up to 600 mmol L^-1^ salt stress (Wang et al., 2014). Therefore, it is an ideal system to analyze the salt tolerance mechanism of woody plants. The root is the first organ injured by salt stress in plants. Therefore, it is important to investigate the proteomic changes of the *K. candel* root under salt stress to reveal its molecular strategies in relation to salt tolerance. In this study, iTRAQ quantitative proteomics was used to analyze protein abundance and expression patterns in *K. candel* seedling roots under high-salinity stress. Enhanced carbohydrate and energy metabolism, antioxidative activity, cell wall structure stability, and metabolism of secondary metabolites in *K. candel* roots were found under salt stress in this study. The findings improve our understanding of the mechanisms of salt tolerance in the woody halophyte.

## 2. Materials and methods

### 2.1. Plant materials and salt treatment

The hypocotyls of *K. candel* were collected from the Zhangjiangkou Mangrove Nature Reserve, Zhangzhou City, Fujian Province (23°55″ N, 117°26″ E). Hypocotyls of similar size and maturity that are free of any form of physical damage, including damages due to pests or disease manifestation, were planted in plastic pots of 45 × 35 × 25 cm. Hoagland nutrient solution was added to the sand culture. The lost water was supplemented every evening, and the nutrient solution was replaced every three days (Wang et al., 2016b). Until the plant grew four leaves (60 days), the seedlings were irrigated with Hoagland nutrient solution without NaCl (control group) and 600 mmol L^-1^ NaCl. The roots of the seedlings were collected 72 h after treatment, and the roots were wrapped in aluminum foil and frozen in liquid nitrogen for 10 min, and then stored at –80 °C for future use. Three biological repeats were performed for each treatment.

### 2.2. Total protein extraction and iTRAQ labeling

Total protein was extracted using a phenol method described by Wang et al. (2014). Approximately 2 g of *K. candel* root was rapidly ground into fine powder in liquid nitrogen, and 10 mL of phenol extraction buffer (0.1 mol L^-1^ Tris, 0.05 mol L^-1^ ascorbic acid (AsA), 0.1 mol L^-1^ KCl, 0.05 mol L^-1^ disodium tetraborate decahydrate, 1% (v/v) Triton X-100, and 2% (v/v) β-mercaptoethanol) were added to the mixture and mixed well. An equal volume of Tris-saturated phenol (pH 8.0) was added and vortexed for 10 min. After centrifugation at 5500 × *g* for 10 min at 4 °C, the phenol layer was transferred to a new tube and mixed with a 6-fold volume of precooled 0.1 mol L^-1^ ammonium acetate methanol solution and precipitated overnight at –20 °C. After centrifugation at 20,000 × *g* for 20 min at 4 °C, the precipitate was suspended in 10 mL of precooled methanol for 1 h at –20 °C. After centrifugation at 20,000 × *g* for 20 min at 4 °C, the precipitate was suspended in 10 mL of precooled acetone with 0.07% β-mercaptoethanol for 1 h at –20 °C. After centrifugation at 20,000 × *g* for 20 min at 4 °C, the step above was performed once more, and the protein powder was stored at –80 °C.

Next, 0.5 mol L^-1^ tetraethylammonium bicarbonate (TEAB) was added and the protein powder was sonicated at 200 W for 20 min, centrifuged at 25,000 × *g* for 20 min at 4 °C, and the supernatant was absorbed. The protein concentration was determined using the Bradford method (Bradford 1976). Typically, 100 µg of protein from each group was added to 5 µg of trypsin and incubated at 37 °C for 12 h. The lysed peptides were then stored in 0.5 mol L^-1^ TEAB. Following the manufacturer’s instructions for the iTRAQ Reagent-8 Plex Multiplex Kit (Applied Biosystems, Foster City, CA, USA), the samples were labeled with tags 114, 115, and 116 for the 600 mmol L^-1^NaCl group and tags 117, 118, and 119 for the control group. Each group contained three biological repeats.

### 2.3. SCX classification and LC-ESI-MS/MS analysis

The labeled peptide solutions were mixed and loaded onto a PolySULFOETHYL 4.6 × 100 mm column (5 µm, 200 Å, PolyLC Inc., Maryland, USA) for gradient elution, followed by desalination with a C18 Cartridge (66872-U, Sigma, Phenomenex, Torrance, CA, USA) and freeze-dried (Unwin et al. 2010).

Peptide solution samples were separated using a nano-high performance liquid chromatography (HPLC) system Easy nLC (Thermo Fisher Scientific, San Jose, CA, USA). The samples were loaded onto a Thermo Scientific EASY column (2 cm × 100 µm, 5 µm-C18), eluted and separated using a Thermo Scientific EASY column (75 µm × 100 mm, 3 µm-C18) at a flow rate of 300 nL min–1. The elution settings were as follows: 0% to 50% B solution (0.1% (v/v) formic acid and 84% (v/v) acetonitrile) for 55 min; 50% to 100% B solution for 2 min; 100% B solution for 3 min.

The samples were separated by HPLC and analyzed using a Q-Exactive mass spectrometer (Thermo Finnigan, San Jose, CA, USA) according to a previous method (Wang et al., 2016b). The analytical procedures were as follows: the time: 60 min; the detection method: positive ions; the scanning range of parent ions: 300–1800 m/z; the resolution of the high-quality MS1 spectra: 70,000 at m/z 200; AGC target: 3e6; the number of scan ranges: 1; and the dynamic exclusion: 40.0 s. The m/z ratios of polypeptide and polypeptide fragments were collected according to the following methods: 10 fragment maps (MS2 scanning) were collected after every full scanning, MS2 activation type: HCD, isolation window: 2 m/z, the resolution of the high-quality MS2 spectra: 17,500 at m/z 200, microscans: 1, normalized collision energy: 30 eV, underfill ratio: 0.1%.

### 2.4. Identification and quantitative analysis of proteins

Mascot 2.2 software (Matrix Science, London, United Kingdom) and Proteome Discoverer 1.4 software (Thermo Fisher Scientic) was used for protein qualitative and quantitative analyses against the *K. candel* transcriptome database (Candel.Unigene.pep.fa, 45215 sequences) (Sandberg et al., 2012). In detail, the raw mass spectrometry data were searched with Mascot 2.2 software against the *K. candel* transcriptome database. The search parameters were: type of search: MS/MS Ion search; enzyme: trypsin; mass values: monoisotopic; max missed cleavages: 2; variable modifications: oxidation (Met) and iTRAQ-8plex (Tyr); peptide mass tolerance: ± 20 ppm; fragment mass tolerance: 0.1 Da. The data were screened according to the standard of FDR < 0.01, and high reliability protein qualitative results were obtained. The quantitation of the proteins was performed using Proteome Discoverer 1.4 software. The protein ratios were calculated as the median of only unique peptides of the protein. All the peptide ratios were normalized by the median protein ratio, and the median protein ratio should be 1 after the normalization. The proteins with fold change ratio of >1.2 or <0.83 and P-value of <0.05 were defined as differentially abundant proteins (DAPs).

### 2.5. Functional classification of the DAPs

Based on three ontologies (cell component, biological process and molecular function), Gene Ontology (GO) function annotation (http://www.geneontology.org) was performed to map the sequences of DAPs. The Kyoto Encyclopedia of Genes and Genomes (KEGG) database (http://www.genome.jp/kegg/or was used for the KEGG pathway analysis of the DAPs. 

### 2.6. Western blot detection of heat shock proteins 70 (HSP70)

Western blot analysis was used to examine the abundance of HSP 70 using the method described by Wang et al. (2014). The sample proteins were separated using 12% SDS-PAGE electrophoresis, and a total of 50 µL of samples were loaded in each well. The proteins in the PAGE were transferred to a nitric acid fiber membrane using the semidry transfer method. Nitric acid fiber membrane was incubated in 0.2 mL cm–2 blocking solution (5% skim milk powder dissolved in TBS buffer (10 mmol L^-1^ Tris-HCl, 150 mmol L^-1^ NaCl, pH 7.5) at room temperature and shaken slowly for 1 h. The nitric acid fiber membrane was washed using TBST buffer (0.05% Tween-20, 10 mmol L^-1^Tris-HCl, and 150 mmol L^-1^ NaCl, pH 7.5). The membrane was further incubated in blocking solution with an HSP 70 antibody (1:5000, Agrisera, Vänäs, Sweden) at room temperature for 1 h. The membrane was washed 3 times with TBST buffer for 5 min each, and it was incubated in alkaline phosphatase-crossed sheep antirabbit antibody (1:10,000, Huamei Bioengineering Company, Shanghai, China) overnight at 4 °C. Afterwards, the membrane was equilibrated in a new buffer (100 mmol L^-1^ Tris-HCl, pH 9.5, 100 mmol L^-1^ MgCl2) and transferred to chromogenic reagent, and the reaction was conducted as described by the manufacturer of the DAB Chromogenic Kit (AR1021, Boster Biological Technology Co., Ltd, Wuhan, China). The intensities of immunoreactive bands were quantified using software Image J (Bethesda, MD, USA), and three independent experiments were performed.

### 2.7. Measurement of physiological and biochemical parameters

The hydroxylamine oxidation (Wang, 1990), titanium tetrachloride (TiCl4) colorimetry (Patterson et al., 1984a) and thiobarbituric acid (TBA) methods (Schmedes and Hølmer 1989) were used to determine the superoxide anion (O_2_ –), hydrogen peroxide (H2O_2_) and malondialdehyde (MDA) contents, respectively. The activities of superoxide dismutase (SOD), peroxidase (POD), catalase (CAT), ascorbate peroxidase (APX), and glutathione reductase (GR) were determined using NBT photoreduction (Hyland et al., 1983), guaiacol (Yu et al., 2011) and Patterson methods (Patterson et al., 1984b), as described by Nakano and Asada (1981) and Halliwell and Foyer (1978), respectively. The glutathione (GSH) and AsA and total triterpene contents were determined according to Law et al. (1983), Guri (1983) and as described in reference (Basyuni et al., 2012), respectively.

The activities of 6-phosphofructokinase (PFK), isocitrate dehydrogenase (IDH), succinate dehydrogenase (SDH), and malate dehydrogenase (MDH) were determined as described by the manufacturer of the corresponding kits (Suzhou Keming Biotechnology Co., Ltd, Suzhou, China). The activities of pyruvate kinase (PK) and pyruvate decarboxylase (PDC) were assayed using the instructions of the kits developed by Nanjing Institute of Bioengineering.

## 3. Results

## 3.1. Changes in the protein abundance of *K. candel* roots in response to high-salinity stress

Using iTRAQ analysis, a total of 286031 secondary mass spectra were obtained in *K. candel* root, of which 50264 were matched with specific peptides. In total, 25,441 peptides were identified, including 22,119 unique peptides, and 5577 proteins were identified. According to the fold change ratio of protein expression, abundance of >1.2 or <0.83 and P-value < 0.05, 227 DAPs were screened. Among them, 110 DAPs were up-regulated and 117 DAPs were down-regulated.

**Figure 1:**
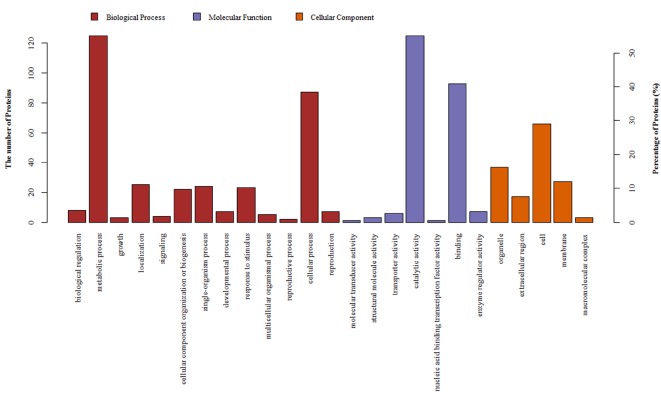
GO classification of differentially abundant proteins identified from *Kandelia candel* roots under high-salinity stress.

The DAPs in the *K. candel* roots that were subjected to NaCl treatment involved three ontologies (cell component, biological processes, and molecular function) as shown in Figure 1. Metabolic processes, cellular processes, and stress responses accounted for a large proportion of the biological processes. Cells, organelles, and cell membranes accounted for a large proportion of the cell composition. Binding molecule function and catalytic activity accounted for a large proportion of the molecular function. Fisher’s exact test was used for GO enrichment analysis of the DAPs. The results showed that the DAPs in the *K. candel* roots subjected to NaCl treatment were significantly enriched in enzymatic activities, carbohydrate metabolism, cell periphery, extracellular area, cell wall, and other functional items (Supplementary Figure 1). These results indicated that salt stress in *K. candel* roots primarily affect biological processes, such as carbohydrate and energy metabolisms and cell structure. Fisher’s exact test was also used for KEGG enrichment analysis and the results showed that the KEGG was primarily enriched in phenylpropane biosynthesis, aminoglycose and nucleotide glycometabolism as well as starch and sucrose metabolism (Supplementary Figure 2).

## 3.2. Functional classifications of the DAPs in *K. candel* roots in response to high-salinity stress

**Figure 2:**
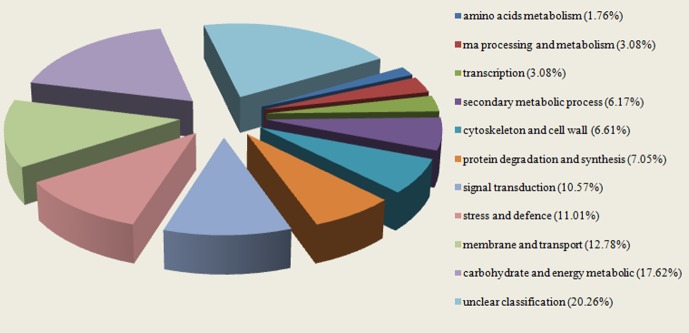
Functional classification of differentially abundant proteins identified from *Kandelia candel* roots under high-salinity stress.

Based on the GO and KEGG analyses, functional classifications of highly reliable DAPs were conducted (Figure 2). The results showed that there were 11 functional classifications of DAPs in *K. candel* roots under NaCl treatment, including carbohydrate and energy metabolisms (17.62%), membrane and transport (12.78%), stress and defense (11.01%), signal transduction (10.57%), protein degradation and synthesis (7.05%), cytoskeleton and cell wall (6.61%), secondary metabolism (6.17%), RNA processing and metabolism (3.08%), transcription (3.08%), amino acid metabolism (1.76%), and unclassified proteins (20.26%). As shown in Table, most of the protein abundances involved in carbohydrate and energy metabolisms, secondary metabolism, stress response and defense, and cell structure were significantly increased, indicating that the *K. candel* root responds actively to these metabolic pathways while under salt stress.

**Table:** Some key differentially abundant proteins from iTRAQ result in *K. candel* roots under high-salinity stress.

Accession	Protein name	Coverage/%	MW/kDa	pI	UP	average 600 mM/0 mM	t-test P-value
Carbohydrate and energy metabolism							
comp19949_c0	UDP-d-glucose 4-epimerase 1	34.2	38.9	7.14	9	1.331	0.020
comp15041_c0	6-phosphofructokinase 3	17.0	61.3	7.08	5	1.736	0.011
CL13463Contig1	Fructokinase 2	25.9	41.4	6.37	5	1.297	0.014
CL12703Contig1	Pyruvate kinase isozyme A	19.0	63.5	5.91	8	1.303	0.010
CL3374Contig1	Pyruvate decarboxylase 2	22.0	65.1	6.28	9	1.799	0.011
CL336Contig2	Pyruvate dehydrogenase kinase isoform 1	32.1	41.6	6.47	8	1.520	0.021
CL8767.Contig2_All	Malate dehydrogenase, mitochondrial (Precursor)	59.9	36.0	8.18	7	1.219	0.012
CL362Contig2	ATP-citrate synthase	30.3	46.7	5.57	7	1.447	0.007
CL4267.Contig4_All	Aconitate hydratase 2	41.6	109.0	7.17	9	1.548	0.015
Unigene3760_All	Phosphoglycerate kinase	66.9	18.5	4.77	6	1.288	0.021
CL10577Contig1	Isocitrate dehydrogenase	51.0	46.9	7.97	9	1.204	0.010
Unigene16086_All	Succinate dehydrogenase 5	40.5	26.5	6.14	6	1.351	0.012
CL8363.Contig1_All	Cytochrome c oxidase subunit 6b-1-like	15.5	21.5	4.35	3	1.204	0.037
Secondary metabolism							
comp22281_c0	1-deoxy-d-xylulose-5-phosphate	3.5	78.0	7.36	1	2.016	0.021
CL8869Contig1	2-c-methyl-d-erythritol -cyclodiphosphate synthase	21.5	25.8	8.57	4	1.219	0.004
comp21082_c0	Flavonoid o-methyltransferase related	8.0	40.1	6.58	2	1.286	0.001
CL773.Contig6_All	2-oxoglutarate fe -dependent dioxygenase-like	11.2	10.8	8.85	1	1.643	0.028
CL5146Contig1	Trehalose-phosphate synthase	10.4	96.6	6.35	4	1.232	0.002
comp30356_c0	Anthocyanidin synthase	7.3	10.9	7.37	1	0.732	0.049
CL11481Contig1	Chalcone synthase	20.8	42.4	6.80	1	0.819	0.003
comp7691_c0	S-adenosylmethionine-dependent methyltransferase	16.7	26.2	6.55	3	1.366	0.006
CL10439Contig1	Myo-inositol oxygenase	22.2	36.5	5.30	7	1.314	0.015
CL7327.Contig2_All	Dihydroneopterin aldolase-like isoform 1	18.8	14.9	7.68	1	1.336	0.020
comp5773_c0	Polyphenol oxidase	39.9	66.7	7.24	19	1.303	0.006
comp20983_c0	Caffeic acid 3-o-methyltransferase	15.8	40.2	5.71	4	0.781	0.008
Stress response and defense							
comp19686_c0	Cationic peroxidase 2	43.7	36.7	8.63	12	1.695	0.004
comp6524_c0	Peroxidase 52-like	46.1	33.9	9.58	11	1.436	0.041
CL3111Contig1	Peroxidase superfamily protein	52.1	36.2	9.72	12	1.894	0.002
CL10686Contig1	Peroxidase superfamily protein	27.1	34.8	9.35	6	1.292	0.010
CL12322Contig1	Peroxidase 47-like	12.3	34.4	5.77	3	0.793	0.036
CL91Contig2	Catalase family protein	43.5	56.9	7.40	13	1.202	0.006
Unigene1381_All	Tau class glutathione transferase gstu52	21.9	17.6	9.11	3	1.247	0.041
comp6225_c0	Glutathione-disulfide reductase isoform 1	51.9	53.5	6.37	19	1.219	0.006
comp6062_c0	OAS-tl3 cysteine synthase	37.9	40.3	8.56	11	1.246	0.011
CL6431.Contig1_All	Cysteine proteinase	69.6	12.6	5.57	10	1.447	0.020
Unigene17872_All	ATP sulfurylase	21.2	51.6	8.53	1	1.398	0.010
Unigene4466_All	S-adenosylmethioninesynthetase	58.3	43.0	6.05	1	0.710	0.007
CL1465Contig1	heat shock protein	18.1	18.4	5.74	1	1.231	0.011
Cell wall and others							
comp5806_c0	Pectinesterase inhibitor 6-like	14.7	58.1	7.69	6	1.432	0.010
CL5352.Contig2_All	Pectate lyase 15-like	5.7	27.2	5.99	1	0.821	0.022
comp10840_c0	Pectin methylesterase family protein	18.0	57.2	9.41	8	0.685	0.035
Unigene2986_All	Xyloglucan endo-transglucosylase hydrolase protein 22	17.4	32.2	7.77	4	0.793	0.021
CL11183Contig1	Endochitinase pr4	38.2	28.6	7.64	9	1.792	0.021

Accession: Database accession numbers according to transcriptome. Coverage: Percent sequence coverage of identified proteins. MW: Theoretical mass (kDa) of identified proteins. pI: pIof identified proteins. UP: Number of unique peptides identified for each protein. average 600 mM/0 mM: average protein abundance of 600 mmol L^-1^ NaCl group vs. 0 mmol L^-1^ NaCl group.

## 3.3. Western blot detection of HSP70

**Figure 3:**
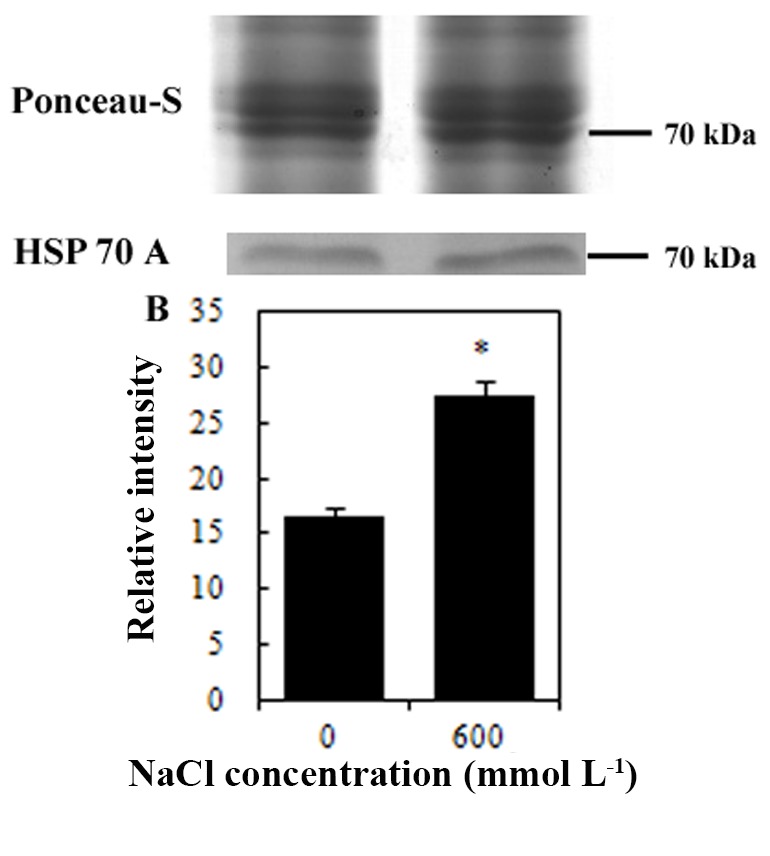
Western blot analysis of HSP 70 in *Kandelia candel* roots under high-salinity stress. Ponceau-S staining was used as loading control. (A) the result of western blot; (B) relative intensity of HSP 70. ‘*’ above the bar indicates a significant difference versus the 0 mmol L^-1^ NaCl group at P < 0.05.

HSP 70 is an important stress responsive protein. In the present study, Western blot assay was used to verify the change in abundance of HSP70 in *K. candel* roots under high-salinity stress (Figure 3). The results showed that the protein abundance of HSP70 increased significantly under the 600 mmol L^-1^ NaCl treatment, which was consistent with the iTRAQ results.

## 3.4. Physiological characteristics of *K. candel* roots in response to high-salinity stress

**Figure 4:**
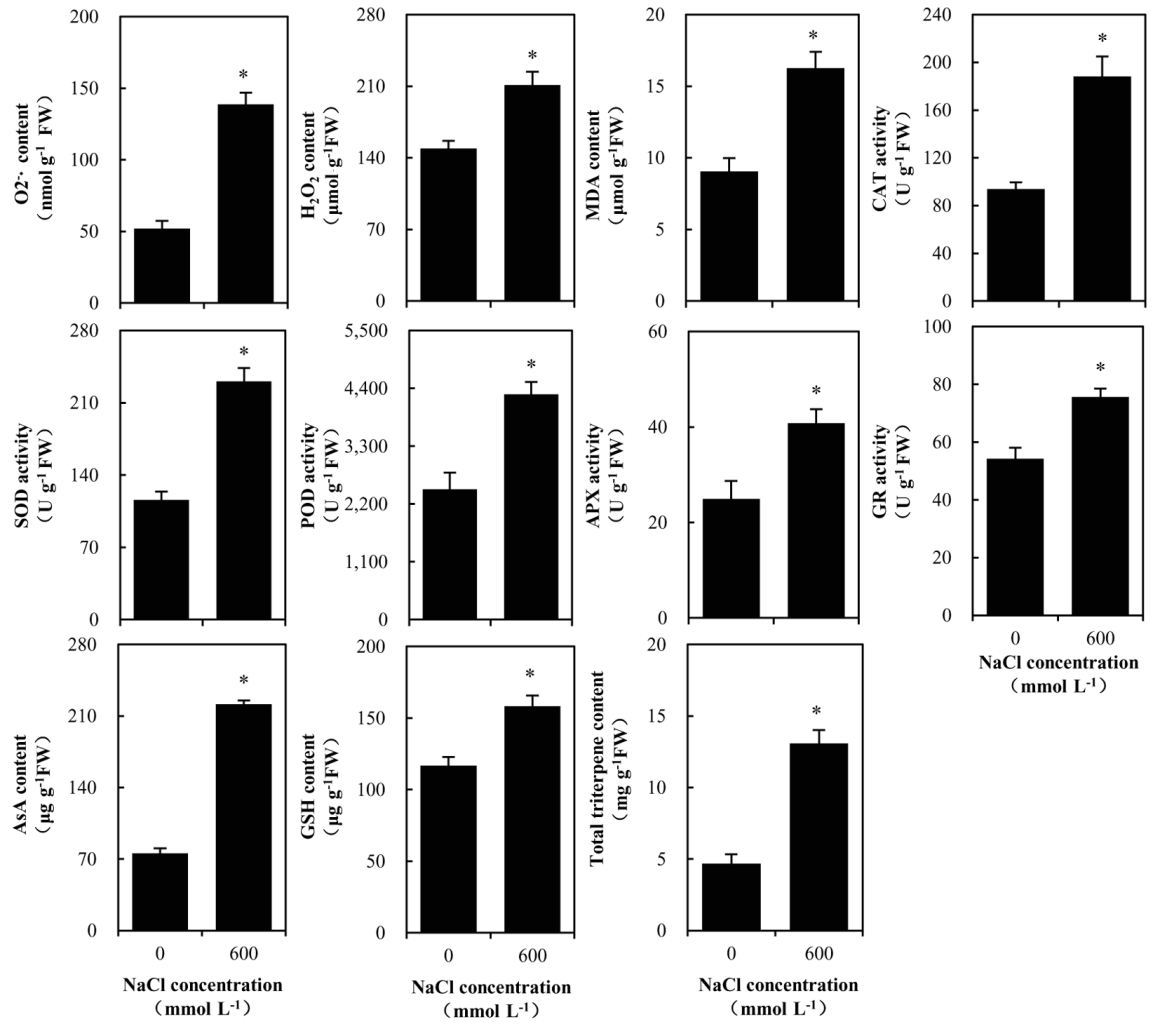
Changes in antioxidative ability and total triterpene content in *Kandelia candel* roots under high-salinity stress. Error bars indicate standard errors of three biological replicates. ‘*’ above the bar indicates a significant difference versus the 0 mmol L^-1^ NaCl group at P < 0.05.

Under the 600 mmol L^-1^ NaCl treatment, the O_2_– and hydrogen H_2_2O_2_ content in the *K. candel* roots was significantly higher than that under the 0 mmol L^-1^ NaCl treatment, while the MDA content increased slightly with no significant difference compared with that under the 0 mmol L^-1^ NaCl treatment (Figure 4). The determination of the activities of antioxidant enzymes and the contents of antioxidant substances in *Kandelia candel* roots showed that the activities of SOD, POD, CAT, APX, and GR, and the contents of antioxidants (GSH and AsA) also increased significantly under 600 mmol L^-1^ NaCl treatment as compared to 0 mmol L^-1^ NaCl treatment (Figure 4). In addition, total triterpenes accumulated to a significant level in *K. candel* roots under high-salinity stress (Figure 4).

**Figure 5:**
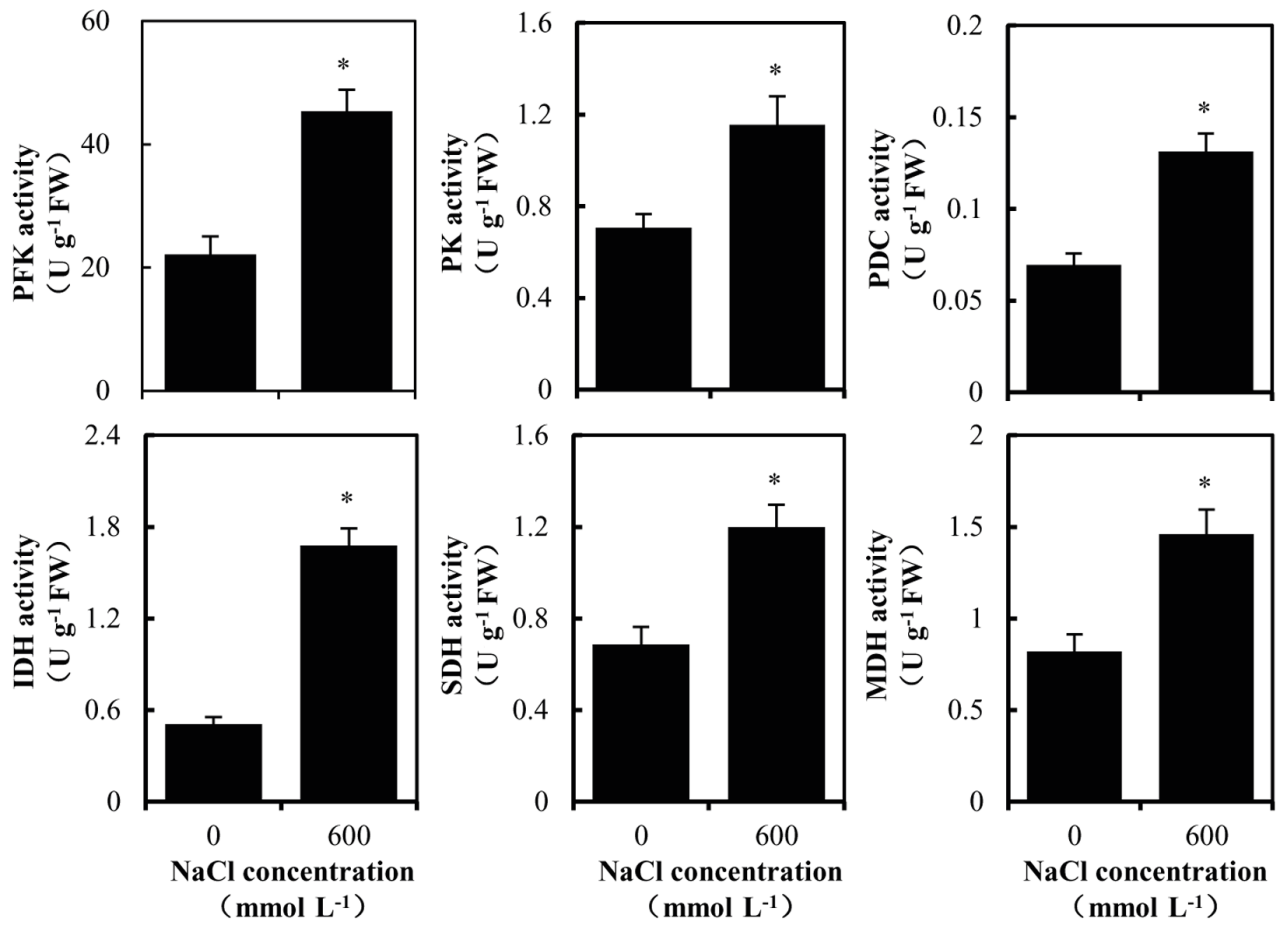
Analysis of EMP-TCA cycle in *Kandelia candel* roots under high-salinity stress. Error bars indicate standard errors of three biological replicates. ‘*’ above the bar indicates a significant difference versus the 0 mmol L^-1^ NaCl group at P < 0.05.

The Embden-Meyerhof-Parnas and tricarboxylic acid (EMP-TCA) pathway is the primary source of energy supply in plants. In this study, the key enzymes in the EMP-TCA pathway were determined and the results are shown in Figure 5. Compared to the 0 mmol L^-1^ NaCl treatment, the activities of PFK, PK, PDC, IDH, SDH, and MDH in the EMP-TCA pathway under 600 mmol L^-1^ NaCl treatment increased significantly.

## 4. Discussion

### 4.1. Carbohydrate and energy metabolisms

It has been found that 9.9%, 21%, and 34.7% of the DAPs participate in carbohydrate and energy metabolisms in response to salt stress in *Raphanus sativus* roots and *Musa paradisiaca* and *A. marina* leaves, respectively (Sun et al., 2017; Shen et al., 2018b; Ji et al., 2019). Previous studies indicated that the effective regulation of protein expression in carbohydrate and energy metabolisms in *H. glomeratus* and *G. hirsutum* roots played important roles in their salt tolerance (Li et al., 2015; Wang et al., 2016a). The roots of the salt-tolerant maize inbred line Jing724 were found to improve in their salt tolerance by enhancing energy supply (Luo et al., 2017). Shen et al. (2018a) reported that salt-tolerant wild barley roots could maintain a normal supply of energy by up-regulating the expression of some key enzymes and proteins involved in the EMP-TCA pathway under salt stress to adapt to a high salt environment. These results suggest that carbohydrate and energy metabolisms play important roles in plant responses to salt stress. Herein, we found that 17.62% of DAPs in *K. candel* roots were involved in carbohydrate and energy metabolisms under high-salinity stress. In plants, the EMP-TCA pathway is the most important biological process in carbohydrate and energy metabolisms. It does not only meet the energy needs of the organism but also provide a variety of intermediates for other metabolic processes (Plaxton and Podestá 2006). The EMP process oxidizes glucose to pyruvate and produces ATP. In this process, PFK, phosphoglycerate kinase (PGK), and PK are the key rate-limiting enzymes. In this study, *K. candel* significantly increased the protein abundances of 6-PFK3 (comp15041_c0), pyruvate kinase isomerase A (CL12703 Contig1), and PGK (Unigene 3760_All) under high-salinity stress (Table 1). The TCA cycle completely oxidizes the pyruvate produced by glycolysis into CO_2_ and generates a large amount of ATP to sustain life (Sweetlove et al. 2010). Citrate synthase (CS), IDH and ɑ-ketoglutarate dehydrogenase are important rate-limiting enzymes in the TCA cycle. Under high-salinity stress, the abundances of pyruvate dehydrogenase kinase (PDK) isomer 1, pyruvate decarboxylase 2 (PDC2), ATP-CS, MDH, IDH, SDH and cytochrome oxidase C (Cyt c) subunits in *K. candel* root increased significantly. The activities of EMP-related enzymes, including PFK, PK, PDC, and the TCA cycle-related enzymes, including PDC, IDH, MDH, and SDH, in *K. candel* roots increased significantly under high-salinity stress, which was consistent with our results of proteome analysis. Therefore, the up-regulation of these important proteins and enzyme activities in the EMP-TCA pathway in *K. candel* roots ensured the normal EMP-TCA pathway and provided sufficient energy for the plant to improve various metabolic processes under a high-salinity environment. Besides the up-regulation of EMP-TCA pathway, increased photosynthesis also plays a crucial role in improving salt tolerance in* K. candel* leaves under salt stress (Wang et al., 2014, 2015, 2016b).

### 4.2. Stress response and defense

Salt stress leads to accumulation of ROS, which leads to the oxidative damage of lipids, proteins and nucleic acids, resulting in the disorder of normal metabolic processes (Parida et al., 2005). Plants can resist ROS-caused oxidative damage by producing various antioxidant enzymes and some other antioxidants (Gill et al., 2010). Previous studies demonstrated that SOD and POD proteins accumulate to high levels in tomato, cotton and wheat under salt stress (Guo et al. 2012; Gong et al. 2014; Li et al. 2015). The up-regulation of proteins related to stress responses and defense in *H. glomeratus* (Wang et al., 2016a) and *G. max *cv Dongnong 50 (Ji et al., 2016) increases their salt tolerance. Furthermore, increased activities of some enzymes related to antioxidant contributed to enhancement of salt tolerance in wheat (*T. aestivum*) roots (Jiang et al., 2017). In this study, our results showed that the protein abundances of four PODs and one CAT in *K. candel* roots increased significantly under high-salinity stress, and the activities of SOD, POD, APX, and CAT also increased significantly. As antioxidants, AsA and GSH participate in the plant resistance to salt stress (Lü et al., 2016). GSH combines with peroxides and free radicals under the action of glutathione transferase (GST) to scavenge excessive ROS and protect the sulfhydryl group of proteins from being destroyed. In transgenic rice and tomato, the accumulation of GST helps the plants adapt to a salt stress environment (Takesawa et al., 2002; Xu et al., 2015b). Under salt stress, the abundance of GST protein in the mangrove *B. gymnorhiza *leaves increases significantly (Zhu et al. 2012). The present study also found that the contents of GST, GSH, and ASA, as well as GR activity increased significantly in *K. candel* roots in response to high-salinity stress. Although the ROS (O_2_– and H_2_2O_2_) content in the *K. candel* roots increased significantly under high-salinity stress, the MDA content did not increase significantly. Similar to the response to salt stress in *K. candel* leaves (Wang et al., 2014, 2015, 2016b), the roots might also initiate antioxidant systems to remove excess ROS and maintain balance of the redox state in the cells under high-salinity stress.

HSPs are stress-responsive proteins which play a crucial role in protecting plants against stress by refolding proteins (Zhu et al., 2012). The up-regulation of HSPs induced by salt stress has been observed in *Physcomitrella patens* (Wang et al., 2008), *Porteresia coarctata* (Sengupta and Majumder 2009), *B. gymnorhiza *(Zhu et al., 2012) and *Medicago sativa* (Xiong et al., 2017). In the present study, the abundance of HSP increased due to 600 mmol L^-1^ NaCl treatment, indicating its protective function to the plant roots during high salinity.

### 4.3. Cell wall structure and stability 

The cell wall is a networked structure composed of polysaccharides, enzymes, and structural proteins, and it is the outermost barrier of plant cells (Micheli 2001). The cell wall first senses stress signals and transduces them into the cells to regulate cellular activities (Kong et al. 2010). Polysaccharides are the primary components of plant cell walls. Changes in the expression of proteins involved in the regulation of cell wall carbohydrates are essential for plants to respond to external stresses. This study found that the protein abundance of xyloglucan endo-transglucosylase hydrolase protein 22 (XTH22) in *K. candel* root was down-regulated, indicating that the *K. candel* root might reduce the decomposition reaction of polysaccharides in the cell wall to maintain the integrity and stability of the cell wall, thereby improving its salt tolerance. Pectin is another major component of plant cell walls. Pectin methylesterase (PME) catalyzes the hydrolysis of pectin methoxy ester to pectic acid and methanol (Lionetti et al., 2007). Balestrieri et al., (1990) found that PME inhibitors form reversible noncovalent complexes with PME in kiwifruit, thus regulating the formation of pectin. We found herein that the expressions of pectin lyase and pectin methylesterase proteins were down-regulated in salt-stressed *K. candel* roots while the expression of a PME inhibitor was up-regulated under the same condition. This indicates that the *K. candel* root enhanced its cell wall stability through accumulation of pectin to resist high-salinity stress.

### 4.4. Triterpenoids and cysteine metabolism

Terpenoids are secondary metabolites in plants and play important roles in plant growth and development, in addition to resistance to abiotic stress (de Costa et al., 2013). In this study, we found that salt stress induced the up-regulation of deoxyketose pentose phosphate (DXS) and 2-C-methyl-D-erythritol cyclophosphate synthase (MEP) proteins related to terpenoid metabolism in *K. candel* roots. Determination of total triterpenoids in the *K. candel* roots showed that the total content of these secondary metabolites increased under high-salinity stress which confirms our results of proteome analysis. Therefore, we postulated that the synthesis of terpenoids might be closely related to the response of *K. candel* roots to high-salinity stress.

Amino acids accumulate in plants under salt stress to protect intracellular nucleic acids, proteins, lipids, and other biomacromolecules from ROS attacks. Moreover, amino acids can also be used as compatible substances to participate in cell osmotic regulation and maintain intracellular pH stability. In this study, we found that the abundances of three cysteine synthesis-related proteins, including cysteine synthase, ATP sulfurylase, and cysteine proteinase, were up-regulated in *K. candel* roots under high-salinity stress. At the same time, cysteine can be desulfurized by the action of L-cysteine desulfhydrase and D-cysteine desulfhydrase, releasing H2S (Calderwood and Kopriva, 2014). As a plant signaling molecule, H2S is involved in multiple biological processes, such as alleviating the damage caused by abiotic stresses such as drought, osmosis, and metal ions (Jin et al., 2011). Therefore, we hypothesize that the up-regulation of cysteine synthesis-related protein in *K. candel* roots might be related to the initiation of H2S signaling pathway in response to high-salinity stress. Furthermore, phenylpropanoids and some free amino acids like γ-aminobutyric acid and glutamate were shown to play an important role in salt tolerance in *K.candel* leaves (Wang et al. 2016b).

In this study, our proteomic and physiological profiles provided important information of salt tolerant regulation network in the woody halophyte *K. candel* roots under 600 mmol L^-1^ NaCl stress for 3 days. We found that carbohydrate and energy metabolisms, stress response and defense, cell wall structure, and secondary metabolites were involved in salt tolerance in *K. candel* roots. Together with the similar physiological profile changes, these findings indicate that *K. candel* roots under high-salinity stress could maintain cell energy supply through up-regulation of the EMP-TCA pathway, regulation of ROS balance by enhancing antioxidation-related enzyme activities and antioxidant contents, stabilizing cell wall structure by reducing polysaccharide decomposition and accumulating pectin, and adjusting cell osmosis through accumulation of triterpenes and other substances. The study expands our knowledge on high-salt adaptation in woody plants and contributes to designing and developing salt-tolerant plants in the future.

## Acknowledgments

This work was supported by the Chinese National Natural Science Funds (Grant no. 331070542), the Natural Science Foundation of Fujian Province, China (Grant no. 2019J01819), Forestry Scientific Research Projects in Public Interest of China (Grant no. 201504415), and the Higher School of Applied Discipline Construction Project of Fujian Province, China (No. 44 document in 2017).
